# The German Version of the Treatment Expectations in Chronic Pain Scale: A Cross-Sectional Validation Study

**DOI:** 10.1155/prm/6612087

**Published:** 2025-09-15

**Authors:** Fabian Rottstädt, Ilona Croy, Lydia Kahle, Kim Ramisch, Winfried Meissner

**Affiliations:** ^1^Department of Clinical Psychology, Friedrich Schiller University Jena, Jena D-07743, Germany; ^2^German Center for Mental Health (DZPG), Site Halle-Jena-Magdeburg, Jena, Germany; ^3^Faculty of Medicine and University Hospital Carl Gustav Carus, TUD Dresden University of Technology, Fetscherstraße 74, Dresden 01307, Germany; ^4^Department of Anesthesiology and Intensive Care Medicine, Jena University Hospital, Friedrich Schiller University of Jena, Jena D-07747, Germany

**Keywords:** chronic pain, ideal expectations, item response theory, Mokken Scale Analysis, realistic expectations, TEC, treatment expectations

## Abstract

**Objective:**

This study aimed to develop and validate a German version of the Treatment Expectations in Chronic Pain Scale (TEC) with the goal to provide a reliable instrument for the assessment of treatment expectations in chronic pain patients within the German healthcare context.

**Methods:**

A total of 153 chronic pain patients participated in the study. Participants were recruited from the outpatient and day clinics of the University Hospital Jena, which specialize in chronic pain treatment. The TEC scale was translated into German following the International Test Commission Guidelines. Psychometric evaluation was conducted using Mokken Scale Analysis, focusing on unidimensionality, scalability, and local independence. For construct validity, correlations were examined with optimism for convergent validity and with depression and anxiety for discriminant validity.

**Results:**

Unidimensionality was supported for the TEC scale overall, but local independence violations were observed for two item pairs on the Ideal Expectations subscale. Furthermore, strong ceiling effects were found in the Ideal Expectations subscale, limiting its discriminatory capacity. Scalability was higher for the Predicted subscale (*H* = 0.475) than for the Ideal subscale (*H* = 0.371). Reliability measures supported the internal consistency. No significant correlations with optimism were found for either subscale, contrary to previous findings.

**Discussion:**

The German TEC displayed a unidimensional structure and is appropriate for group-level analyses of treatment expectations. For individual comparisons, the Predicted subscale offers sufficient precision. Future studies with larger, more diverse samples should confirm these results and clarify how expectations shape adherence and outcomes.

**Trial Registration:**

German Clinical Trials Register (DRKS): DRKS00027071

## 1. Introduction

In chronic pain therapy, patients' expectations play a crucial role in shaping treatment outcomes, influencing factors such as satisfaction, adherence, and even the degree of symptom improvement. Research highlights that patients with positive treatment expectations tend to experience greater improvements in symptoms and quality of life than those with lower expectations [1, 2]. This relationship is attributed, in part, to psychological mechanisms similar to the placebo effect, where positive expectations foster beneficial cognitive and emotional responses that enhance treatment efficacy [3]. Furthermore, for medical treatment, it has been shown that negative expectations, concerns, and fears about a treatment can intensify unwanted side effects [4, 5]. The findings suggest that expectations for pain therapy can positively or negatively influence the outcome. Consequently, assessing treatment expectations is an important aspect of optimizing therapy outcomes.

In the context of chronic pain, treatment expectations play an especially crucial role due to the often long-term and fluctuating nature of symptoms. Positive expectations have been shown to shape treatment effects in cases of chronic pain [6–8]. It has also been shown that interventions that shape expectations can provide pain relief, leading to small effects on chronic pain [9]. A pilot study on interdisciplinary multimodal pain therapy (IMPT) for chronic back pain demonstrated that positive treatment expectations correlate with better therapeutic outcomes. Patients with higher expectations showed significant improvements in pain intensity and functional impairment [10]. Patients with chronic pain frequently experience multiple treatment attempts with varying degrees of success, which can influence their expectations and treatment engagement over time [11]. On the other hand, unrealistically high expectations, especially if unmet, can lead to dissatisfaction, frustration, and reduced adherence, further complicating treatment and potentially worsening pain-related disability. Realistic yet positive expectations, however, may help patients maintain motivation, manage their symptoms more effectively, and adapt to long-term therapy goals.

Treatment expectations can be categorized into three primary types: structural, process, and outcome expectations [12]. Structural expectations involve beliefs about the physical environment, available resources, and qualifications of healthcare providers (e.g., “The specialist treating me is highly qualified”). Process expectations reflect patients' beliefs about the methods and care they will experience during treatment, such as empathy and communication from providers (e.g., “The pain specialist will understand my challenges”). Outcome expectations focus on the anticipated results of treatment, including potential pain reduction and functional improvement (e.g., “My pain will significantly decrease”).

To systematically measure these expectations, Pagé et al. [13] developed the Treatment Expectations in Chronic Pain Scale (TEC), specifically targeting outcome and process expectations. The TEC scale provides a comprehensive understanding of patients' hopes and beliefs regarding the therapeutic process and potential results. It is structured into two subscales: ideal expectations (what patients hope to achieve under optimal conditions) and predicted (realistic) expectations (what patients actually anticipate will happen during treatment). This distinction was made under the assumption that a large gap between ideal and realistic expectations may lead to dissatisfaction or reduced adherence if expected results are not achieved. The TEC scale was designed using item response theory. Psychometric analyses of the original TEC scale demonstrated high internal consistency (Cronbach's *α* ≈ 0.87–0.88), medium scalability (Loevinger's *H* ≈ 0.48), and no violations of monotonicity or local independence once the Ideal and Predicted facets were analyzed separately. Correlations with depression and anxiety were very small (|*r*| ≤ 0.14), correlation with optimism were low (*r* = 0.24) [13].

Chronic pain is highly prevalent, affecting an estimated 27% of the general adult population in Europe [14], with around 17% of people in Germany affected [15]. The impact on individuals is profound, as it not only reduces quality of life but also restricts daily functioning and productivity [16], leading to significant socioeconomic consequences [17].

Due to the high prevalence and significant impact of chronic pain in Germany, a validated instrument for assessing treatment expectations seems important. This study aims to validate the German version of the TEC scale, to provide a tool for the standardized assessment of treatment expectations in chronic pain therapy within the German healthcare system.

## 2. Materials and Methods

### 2.1. Sample and Study Design

We carried out a cross-sectional psychometric validation of the TEC scale. All participants were receiving treatment because of chronic pain conditions in either the Outpatient Clinic for Pain Patients or the Day Clinic for Pain Patients at the University Hospital in Jena. Patients were invited to complete the TEC scale, in addition to the routine clinical questionnaires administered during their treatment, after providing written informed consent. Participants had to be (1) 18 years of age or older, (2) diagnosed with chronic pain, (3) currently receiving outpatient, day-clinic (partial-inpatient), or inpatient treatment at the study center, and (4) sufficiently proficient in German to complete the study questionnaires. All consecutively attending chronic pain patients who met the eligibility criteria during the recruitment period were approached, irrespective of diagnosis subtype or treatment modality.

Sample characteristics and diagnostic overview can be found in Table 1. A total of 192 patients with chronic pain initially participated in this study by completing the TEC questionnaire. Among these, 39 patients who did not complete the TEC scale were fully excluded from the analysis to ensure data integrity and accuracy. The final sample consisted of 153 patients. The sample was characterized by a mean age of 55.3 years (SD = 15), and the majority of participants were female (69.3%). Participants were required to be at least 18 years old and have sufficient German language skills. Participation was voluntary and without compensation.

Diagnoses varied widely and included back pain, neuropathic pain, and musculoskeletal disorders, reflecting the clinical diversity commonly observed in pain treatment settings. Based on the first three coding positions of the ICD-10-GM [18], the sample included over 100 different diagnoses.

Musculoskeletal and connective tissue diseases (M diagnoses) were recorded at least once in 128 patients (66.3%). The three most frequent diagnoses were dorsalgia (M54; *n* = 80; 41.5%), other soft tissue disorders, not elsewhere classified (M79; *n* = 57; 29.5%), and other joint disorders, not elsewhere classified (M25; *n* = 46; 23.9%). Nervous system diseases (G diagnoses) were recorded at least once in 86 patients (44.5%). The three most frequent diagnoses were other polyneuropathies (G62; *n* = 42; 21.7%), other headache syndromes (G44; *n* = 29; 15.0%), and migraine (G43; *n* = 14; 7.3%). Mental and behavioral disorders (F diagnoses) were recorded at least once in 99 patients (51.3%). The most common diagnosis was somatoform disorder (F45; *n* = 91; 47.2%), followed by recurrent depressive disorder (F33; *n* = 15; 7.8%), and other anxiety disorders (F41; *n* = 4; 2.1%). An overview of all recorded diagnoses can be found in the supporting information (see supporting 2).

On the von Korff scale, the majority of patients (*N* = 98; 34.9%) reported Grade 4, indicating severe disability and high pain levels. Grade 3 (high pain and moderate disability) was reported by *N* = 46 patients (16.4%). Grades 1 (*N* = 10; 3.6%) and 2 (*N* = 21; 7.5%) were reported rarely. Overall, the sample demonstrated a high burden of pain and disability.

### 2.2. Ethics Statement

The study followed the Declaration of Helsinki for medical research involving human subjects and was approved by the Ethics Committee of the University Hospital Jena (Reg.-Nr.: 22/098). All participants provided written informed consent.

### 2.3. Methods

#### 2.3.1. Outcome Measures

##### 2.3.1.1. TEC

The TEC by Pagé et al. [13] assesses treatment expectations in patients with chronic noncancer pain. This nine-item self-report questionnaire includes five items targeting outcome expectations and four items addressing process expectations. Each item is answered twice: once under an ideal scenario (ideal expectations) and once under a real-world scenario (predicted expectations). Respondents indicate their level of agreement on a five-point scale (1 = strongly disagree to 5 = strongly agree). The sum scores for each scenario represent the participant's ideal and predicted expectations, while the difference between these scores reflects the discrepancy between the respondent's wishes and their realistic expectations. However, Pagé et al. [13] suggest interpreting the two scores independently.

For this study, the TEC scale was translated into German following the International Test Commission Guidelines for Translating and Adapting Tests [19]. The translation process included a forward and backward translation: the English source text was first translated into German by a native German speaker, and this German version was then translated back into English by a native English speaker. Both versions were compared to identify and resolve discrepancies, leading to the development of a final version. The translation was carried out by a certified translator in accordance with the EN ISO 17100 standard. The translated version, used in this research, is provided in Supporting 1.

##### 2.3.1.2. The Life Orientation Test-Revised (LOT-R)

The LOT-R is a self-report instrument developed by Scheier, Carver, and Bridges [20] to assess individual differences in generalized optimism versus pessimism as a unidimensional personality trait. It consists of 10 items: three measuring optimism, three measuring pessimism, and four filler items. Participants rate their agreement with each item on a five-point scale (0 = does not apply at all to 4 = is very true). To calculate the final score, pessimism items are reverse-coded and summed with optimism items. For the German version of the LOT-R, it is recommended to calculate separate sum scores for optimism and pessimism [21].

##### 2.3.1.3. German Pain Questionnaire (GPQ) (Deutscher Schmerzfragebogen)

The GPQ is a modular instrument for assessing chronic pain [22]. In this study, three specific modules of the GPQ were utilized: Depression, Anxiety, and Stress Scales (DASS), the von Korff scale, and pain duration. These modules are described below.

###### 2.3.1.3.1. DASS

It is a screening tool for assessing depression, anxiety, and stress in individuals with chronic pain. It consists of 21 items, with seven items per scale. Respondents rate how well each statement applied to them over the past week on a four-point scale (*“Did not apply to me at all”* = 0 to *“Applied to me very much or most of the time”* = 3). The scores for each subscale are calculated by summing the item responses [23]. The German version has shown good internal consistency: depression (*α* > 0.91), anxiety (*α* = 0.78–0.82), and stress (*α* = 0.81–0.89). Strong convergent validity was observed, with correlations of *r* = 0.68 (depression scale with Beck Depression Inventory) and *r* = 0.76 (anxiety scale with Beck Anxiety Inventory) [23].

###### 2.3.1.3.2. von Korff Scale

Part of the Graded Chronic Pain Scale (GCPS) measures chronic pain severity across two dimensions: pain intensity and pain-related disability. The scores for intensity and disability are combined to classify patients into five severity grades: Grade 0 (no pain or disability), Grade 1 (low pain intensity, minimal disability), Grade 2 (high pain intensity, low disability), Grade 3 (moderate to severe disability), and Grade 4 (severe disability affecting multiple life areas). The von Korff scale has demonstrated acceptable internal consistency (*α* = 0.67–0.74) and strong convergent validity, with significant correlations to depression and medication use [24].

###### 2.3.1.3.3. Pain Duration

The duration of pain was assessed using the question *“Since when has the pain existed?”*, which was answered on a six-point scale ranging from *“less than 1 month”* to *“more than 5 years*.*”*

### 2.4. Statistical Analysis

The TEC scale was analyzed using the procedure for Mokken Scale Analysis outlined by Sijtsma and Van der Ark [25] utilizing a monotone homogeneity model, implemented with the R package *Mokken* [26] in R Version 4.0.3 with the following steps. (1) Automated Item Selection Procedure (AISP) was applied to assess scale identification. The AISP procedure was run for positive constant c initially set at 0.0 and then rerun with c increased by increments of 0.05 until reaching *c* = 0.55. (2) Loevinger's H coefficient was calculated for individual items and subscales to assess scalability. (3) Item pairs were assessed for violations of local independence using *ω*^1^ and *ω*^3^ indices. (4) Monotonicity violations were tested using nonparametric regression and graphical analysis. Reliability of the scales was assessed using Cronbach's alpha, Guttman's lambda^2^, and McDonald's omega.

Following the methodology of Pagé et al. [13], the construct validity of the TEC scale was assessed using optimism, measured with the LOT-R, for convergent validity, and depression, anxiety, and stress, measured with the DASS, for discriminant validity (for internal consistency of those scales, please compare Supporting 4). Pearson correlations were calculated to evaluate these relationships. In contrast to the study by Pagé et al. [13], the total sum score of the LOT-R combining optimism and pessimism items was not used. Instead, only the sum score of the optimism items was included in the analysis following recommendations by Herzberg et al. [21]. Furthermore, Pearson correlation analyses were conducted to examine the association between expectations and age, while independent *t*-tests were performed to assess gender differences in expectations (significance level was set at *α* = 0.05).

## 3. Results

### 3.1. Mokken Scale Analysis

1. AISP was applied to identify items that form unidimensional scales. Initial scalability checks revealed that items I1 and I3 should be removed due to low Loevinger's H coefficients (*H* < 0.3). After excluding these items, the remaining items were assessed for unidimensionality. Using the default threshold value of *c* = 0.3, unidimensionality was observed, as described by Sijtsma and van der Ark. When the c-value was gradually increased from 0 to 0.55 in increments of 0.05, the same pattern emerged, providing further evidence of unidimensionality for the remaining items. The subscales “Ideal Expectations” and “Predicted Expectations” could not be distinctly identified in the German translation of the TEC scale.
2. Loevinger's H was calculated for individual items and subscales to assess scalability. The H-coefficient for the ideal subscale was *H* = 0.371, and that for the predicted subscale was *H* = 0.475. The overall scalability of the TEC scale was *H* = 0.372, indicating a weak scale.
3. Two item pairs violated the assumption of local independence: I3 with I5 and I9 with R1. These violations suggest potential redundancy or overlap in the content of these items.
4. No significant violations were detected for any items, suggesting that all items adhered to the assumption of monotonicity. Graphical analyses were also conducted to test for violations of monotonicity. Those item response functions for all items are shown in Supporting 3

### 3.2. Scale Properties

Cronbach's alpha, Guttman's lambda^2^, and the corrected item-total correlation and H coefficients are presented in Table 2. The removal of any single item did not improve reliability (*α* = 0.786–0.862). McDonald's omega was calculated for the overall TEC scale (*ω*_*h*_ = 0.89, *ω*_*t*_ = 0.89) and both subscales separately (ideal subscale: *ω*_*h*_ = 0.83 and *ω*_*t*_ = 0.83; predicted subscale: *ω*_*h*_ = 0.87 and *ω*_*t*_ = 0.87). These results indicate that the TEC scale and its subscales exhibit acceptable reliability.

**Figure 1:**
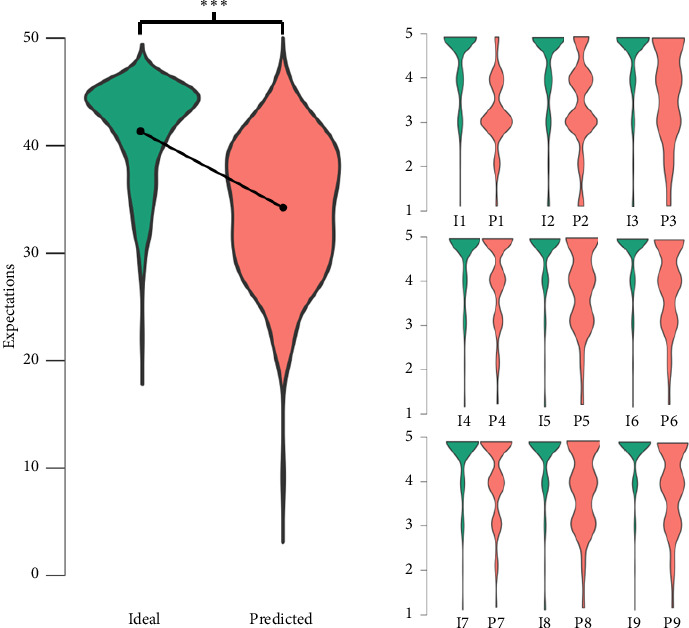
Violin plots show the distribution of scores for each subscale and item of the TEC scale indicating that ideal expectations scores were consistently higher than predicted expectations scores; I 1–9 and P 1–9 represent the 9 items of the Ideal Expectations (I) and Predicted Expectations (P) subscales; dots indicate mean; ^∗∗∗^significant difference between subscales with *p* < 0.001.

Distribution of scores for each subscale can be found in Figure 1 and Table 3. A ceiling effect was found for the items on the ideal subscale, with 62%–78% of participants selecting the highest score, and for item P4, with 51% selecting the highest score. Responses on the Ideal Expectations scale were significantly higher than those on the Predicted Expectations scale (*z* = −10.34, *p* < 0.001).

### 3.3. Construct Validity

Neither the overall TEC nor the Ideal Expectations or the Predicted Expectations subscale showed significant correlations with a measure of optimism. There were also no significant correlations between the subscales and measures of anxiety and depression. In Table 4, results of the correlation analyses are shown. In the analysis of the two subscales, no significant gender differences were observed in the responses (overall TEC scale: *t* (153) = 0.97, *p*=0.34; Ideal Expectations: *t* (153) = 0.19, *p*=0.85; Predicted Expectations: *t* (153) = 1.04, *p*=0.30). There were also no correlations between both subscales and age (Ideal Expectations: *r* = 0.02, *p*=0.85; Predicted Expectations: *r* = 0.13, *p*=0.12).

## 4. Discussion

The validation of the German version of the TEC aimed to assess its psychometric properties. Overall, the German TEC scale was found to have a unidimensional structure and provides a global assessment of treatment expectations, combining ideal and predicted expectations. In this sample, the TEC (*H* = 0.37) meets the basic criterion (*H* ≥ 0.30) for forming a coherent scale [25], making its total score appropriate for analyses at the group level, where comparisons of group means can be made with reasonable confidence [27]. The instrument sits just below the lower boundary for individual-level interpretation (*H* ≥ 0.40); small score differences between single patients should therefore be viewed with caution. Reliability was high, demonstrating strong internal consistency for the overall scale, and the absence of meaningful correlations with anxiety or depression supports its discriminant validity. Analyses revealed no evidence of gender- or age-related differences in TEC scores, suggesting metric stability across these demographic groups.

To maintain continuity with the original validation study and to explore whether facet-specific scores might still offer distinct clinical insight, we subsequently analyzed the Ideal and Predicted item sets separately. In terms of scalability, the Predicted subscale demonstrated moderate scalability (*H* = 0.48) allowing for differentiation between individuals, while the Ideal subscale demonstrated weaker scalability (*H* = 0.37), making its total score appropriate for analyses at the group level. Another notable characteristic of the Ideal subscale is the presence of strong ceiling effects that limit its ability to differentiate between individuals with high ideal expectations, reducing its discriminatory capacity [28]. Both subscales—like the overall TEC scale—showed negligible correlations with anxiety, depression, or dispositional optimism, supporting discriminant validity, and each exhibited satisfactory internal consistency.

### 4.1. Recommendations for Using the German TEC Scale

The German version of the TEC can be found in Supporting 1. Taken together, these findings support a two-tiered use of the German TEC [29]. At the broadest level, the instrument functions as a single, coherent measure of treatment expectations in chronic pain and can be applied with confidence whenever a global expectancy index is sufficient—for example, in research that compares groups. In such cases, the overall sum of the TEC scale can be computed by adding the answers of all items, both of the Ideal and Predicted subscales, with higher values indicating more positive overall treatment expectations.

When greater precision or minimal administration time is needed—for example, in routine clinical practice—the nine-item Predicted subscale offers a reliable stand-alone option. Its solid psychometric profile enables clinicians to quickly gauge patients' realistic treatment expectations and distinguish individual differences.

### 4.2. Comparison to Original TEC Scale

The validation of the German version of the TEC revealed both similarities and differences when compared to the original TEC scale developed by Pagé et al. [13]. Both the German and the original versions of the TEC scale met the assumption of monotonicity, showed good internal consistency, exhibited strong ceiling effects for the Ideal Expectations subscale, and showed low correlations with anxiety and depressive symptoms, supporting the assumption that treatment expectations are conceptually distinct from general emotional distress. However, Pagé et al. [13] identified two distinct subscales: Ideal Expectations and Predicted Expectations. In contrast, in the German version, the two subscales were less distinct, as the items from both subscales did not group as clearly. Furthermore, convergent validity of the English version was supported by a significant positive correlation with optimism (LOT-R). In contrast, no significant correlations were observed between optimism and either the Ideal or Predicted Expectations subscales in the German version. It is important to note that the German version of the LOT-R exhibits a different factor structure than the English version, which could contribute to the differing results. Item-level scalability in the German TEC was generally lower than that in the English original, with H-coefficients ranging from 0.25 to 0.46; nevertheless, no violations of monotonicity were observed for any item. Reliability mirrored the pattern reported by Pagé et al.: the Predicted subscale reached an equally high internal consistency, whereas the Ideal subscale showed a slightly reduced—but practically comparable—value.

The different findings between the two studies may be attributed to several factors. One key consideration is the composition of the sample. As the work by Pagé et al. does not report the chronic burden of the included participants, it is not possible to compare the two samples in terms of illness severity. One difference can be identified as Pagé et al.'s sample included patients on a waitlist for a multidisciplinary pain treatment center, while the German version relied on patients who were already undergoing treatment in outpatient and day clinics within specialized chronic pain treatment centers. Patients already in treatment may have different expectations, which could influence how they conceptualize and respond to items about ideal and predicted treatment outcomes. Translation nuances could also contribute to the observed differences. Subtle differences in the meaning of words or statements between the German and English versions may have led to greater conceptual overlap between the subscales. Furthermore, the ability to detect distinct subscales depends on the sample size and statistical power. Compared to Pagé et al.'s study, the German version is based on a smaller sample size. A smaller sample can reduce the power of the AISP to detect distinct subscales, especially if the conceptual differences are subtle.

### 4.3. Comparison to Other Expectancy Questionnaires

Compared with other validated expectation measures, the TEC occupies a distinctive middle ground. It is substantially more concise than the multidimensional TEX-Q [30] and the EXPECT [31] questionnaire, yet—unlike the ultra-brief ETS [32] and the valence-oriented SETS [33]—it captures not only outcome expectations but also process expectations, thereby offering a broader view of how patients anticipate their treatment experience. Moreover, while none of these comparators was developed expressly for chronic pain populations, the TEC was tailored to this context and therefore targets clinically relevant goals beyond pain reduction—such as routine activities, mood, knowledge, and sleep—that frequently guide treatment decisions in chronic pain management. Taken together, the TEC complements the existing expectancy measure toolkit and fills a niche for a short, chronic pain–specific instrument that combines outcome and process-focused content.

## 5. Limitations

The sample for the German version is based on a specific sample composition which may limit the generalizability of the findings, as it does not reflect the broader population of primary care patients or outpatients in general pain clinics. Patients in specialized pain clinics often have higher illness severity and more complex treatment histories [34–36], which may influence their treatment expectations. To ensure broader applicability, future studies should validate the TEC scale in more diverse samples. This would allow for a better understanding of how the psychometric structure of the German TEC scale differs across less burdened and more treatment-naive patient populations. The smaller sample size in the German version compared to Pagé et al. [13] may have limited the statistical power of the AISP, making it more difficult to detect two distinct subscales [25, 37, 38].

The present study used a cross-sectional design. As a result, key psychometric properties, such as test-retest reliability, could not be assessed. Future research should adopt longitudinal study designs to track changes in patient expectations over the course of treatment and examine how these changes relate to treatment satisfaction, adherence, and clinical outcomes.

## 6. Conclusion

Treatment expectations are a key determinant of adherence, satisfaction, and therapeutic engagement [6–8, 11], so routinely assessing them benefits both research and clinical care by supporting patient-centered, expectation-aligned interventions and shared decision making. The German TEC, which displays a unidimensional structure, serves as a reliable global expectancy index for group-level comparisons and program evaluation in research settings. For work with individual patients, the Predicted Expectations subscale offers a concise tool for capturing realistic treatment outlooks, enabling clinicians to identify and address misaligned expectations early in the care process.

## Figures and Tables

**Table 1:** Sample characteristics and diagnostic overview.

Characteristic	*n*	%	M	SD
Final sample	153	100		
Female participants	106	69.3		
Age (years)	—	—	55.3	15.0

*Diagnosis group (ICD-10)*				
Musculoskeletal diseases (M)	128	66.3		
M54—dorsalgia	80	41.5		
M79—other soft tissue disorders	57	29.5		
M25—other joint disorders	46	23.9		
Nervous system diseases (G)	86	44.5		
G62—other polyneuropathies	42	21.7		
G44—other headache syndromes	29	15.0		
G43—migraine	14	7.3		
Mental and behavioral disorders (F)	99	51.3		
F45—somatoform disorder	91	47.2		
F33—recurrent depressive disorder	15	7.8		
F41—other anxiety disorders	4	2.1		
von Korff pain grade	n	%		
Grade 1 (low disability, low pain)	10	3.6		
Grade 2 (low disability, high pain)	21	7.5		
Grade 3 (high pain, moderate disability)	46	16.4		
Grade 4 (high pain, severe disability)	98	34.9		

*Note:* Diagnoses were coded according to the ICD-10-GM [18], using the first three digits. For every chapter, only the three most frequent diagnoses are reported in the table. A complete list of diagnoses is provided in Supporting 2. Patients could have more than one diagnosis.

**Table 2:** Reliability of the German Treatment Expectations in Chronic Pain Scale.

	Corrected item-total correlation	Cronbach's alpha if item deleted	Cronbach's alpha/Guttman's lambda^2^	*H*-coefficient
Complete TEC scale	0.89/0.88	0.37		
Predicted subscale	0.87/0.87	0.48		
R1. My pain will be significantly reduced	0.53	0.86		0.32
R2. I will be able to do routine activities (cooking, cleaning, self-hygiene) better	0.60	0.86		0.43
R3. I will learn the reason for my pain	0.57	0.86		0.36
R4. I will receive a clear pain treatment plan	0.70	0.85		0.45
R5. I will learn about ways I can manage my pain condition	0.57	86		0.38
R6. I will learn more about my pain condition	0.58	0.86		0.36
R7. My mood will significantly improve	0.67	0.85		0.46
R8. The pain specialist will understand my situation and all its challenges	0.62	0.85		0.41
R9. My sleep will significantly improve	0.62	0.85		0.42
Ideal subscale	0.82/0.83	0.37		
I1. My pain will be significantly reduced	0.40	0.81		0.27
I2. I will be able to do routine activities (cooking, cleaning, self-hygiene) better	0.62	0.79		0.37
I3. I will learn the reason for my pain	0.38	0.82		0.25
I4. I will receive a clear pain treatment plan	0.60	0.79		0.45
I5. I will learn about ways I can manage my pain condition	0.53	0.80		0.34
I6. I will learn more about my pain condition	0.55	0.80		0.35
I7. My mood will significantly improve	0.64	0.79		0.38
I8. The pain specialist will understand my situation and all its challenges	0.53	0.80		0.32
I9. My sleep will significantly improve	0.50	0.81		0.30

*Note:* Corrected item-total correlations, Cronbach's alpha if item deleted, Cronbach's alpha/Guttman's lambda^2^, and H-coefficients for the complete scale and the subscales “Predicted” and “Ideal.”

**Table 3:** Scale properties.

	M	(SD)	[Range]	*N* and percentage of patients who selected that answer				
				1 (strongly disagree)	2 (disagree)	3 (neutral)	4 (agree)	5 (strongly agree)
Predicted subscale	33.9	(6.2)	[9–45]					
R1.	3.2	(0.8)	[1–5]	4 (2.6%)	14 (9.2%)	86 (56.2%)	39 (25.5%)	10 (6.5%)
R2.	3.5	(1.1)	[1–5]	9 (5.9%)	12 (7.8%)	58 (37.9%)	48 (31.4%)	26 (17.0%)
R3.	3.8	(1.1)	[1–5]	6 (3.9%)	13 (8.5%)	39 (25.5%)	46 (30.1%)	49 (32.0%)
R4.	4.2	(1)	[1–5]	1 (0.7%)	9 (5.9%)	25 (16.3%)	40 (26.1%)	78 (51.0%)
R5.	3.9	(1)	[1–5]	4 (2.6%)	6 (3.9%)	46 (30.1%)	48 (31.4%)	49 (32.0%)
R6.	4	(0.9)	[1–5]	2 (1.3%)	7 (4.6%)	34 (22.2%)	54 (35.3%)	56 (36.6%)
R7.	3.7	(0.9)	[1–5]	2 (1.3%)	12 (7.8%)	52 (34.0%)	56 (36.6%)	31 (20.3%)
R8.	3.8	(1)	[1–5]	2 (1.3%)	14 (9.2%)	43 (28.1%)	43 (28.1%)	51 (33.3%)
R9.	3.7	(1.1)	[1–5]	6 (3.9%)	11 (7.2%)	52 (34.0%)	40 (26.1%)	44 (28.8%)
Ideal subscale	41.2	(4.5)	[22–45]					
I1.	4.6	(0.7)	[2–5]	0 (0)	1 (0.7%)	18 (11.8%)	26 (17.0%)	108 (70.6%)
I2.	4.4	(1)	[1–5]	5 (3.3%)	2 (1.3%)	16 (10.5%)	27 (17.6%)	103 (67.3%)
I3.	4.6	(0.9)	[1–5]	4 (2.6%)	2 (1.3%)	11 (7.2%)	23 (15.0%)	113 (73.9%)
I4.	4.6	(0.8)	[1–5]	1 (0.7%)	2 (1.3%)	13 (8.5%)	21 (13.7%)	116 (75.8%)
I5.	4.5	(0.9)	[1–5]	5 (3.3%)	2 (1.3%)	7 (4.6%)	29 (19.0%)	110 (71.9%)
I6.	4.7	(0.6)	[3–5]	0 (0)	0 (0)	8 (5.2%)	25 (16.3%)	120 (78.4%)
I7.	4.4	(0.8)	[1–5]	1 (0.7%)	5 (3.3%)	14 (9.2%)	38 (24.8%)	95 (62.1%)
I8.	4.6	(0.7)	[2–5]	0 (0)	3 (2.0%)	13 (8.5%)	24 (15.7%)	113 (73.9%)
I9.	4.5	(1)	[1–5]	3 (2.0%)	6 (3.9%)	14 (9.2%)	19 (12.4%)	111 (72.5%)

*Note:* Means, standard deviations, observed ranges, and response frequencies for all items of the Predicted and Ideal subscales. Percentages reflect the proportion of patients selecting each response option (1 = strongly disagree to 5 = strongly agree).

**Table 4:** Spearman's rank correlation coefficients between the German Treatment Expectations in Chronic Pain subscales and measures of optimism, anxiety, and depression.

	TEC_Sum	TEC_P	TEC_I	LOT-R-O	DASS-D	DASS-A
TEC_Sum	75.14 (9.3)					
TEC_P	0.93^∗∗^	33.9 (6.2)				
TEC_I	0.73^∗∗^	0.46^∗∗^	41.2 (4.5)			
LOT-R-O	−0.06	−0.04	−0.10	6.3 (3.3)		
DASS-D	0.05	0.004	0.15^∗∗^	−0.07	8.2 (5.4)	
DASS-A	−0.09	−0.12	0.004	0.09	0.50^∗∗^	4.9 (4.6)

*Note:* Means and standard deviations are shown in the diagonal. TEC_Sum: overall sum of the TEC; TEC_P: TEC Predicted Expectations subscale; TEC_I: TEC Ideal Expectations subscale; TEC_Diff: difference between ideal and predicted subscale score; LOT-R: Life Orientation Test-Revised, optimism scale; DASS-D: depression scale of the Depression, Anxiety, and Stress Scale; DASS-A: anxiety scale of the Depression, Anxiety, and Stress Scale.

^∗∗^ *p* < 0.01.

## Data Availability

The data that support the findings of this study are available from the corresponding author upon reasonable request.

## Supporting Information

Supporting information is provided for the online version of the article (attached to article). It contains the German version of the TEC that was used in this research (Supporting 1), an overview of all recorded diagnoses in the sample (Supporting 2), the item response functions for all items (Supporting 3), and internal consistencies of all additional measures (Supporting 4).
