# Supplementary material for: The German Version of the Treatment Expectations in Chronic Pain Scale: A Cross-Sectional Validation Study
Source: Pain Res Manag. 2025 Sep 15;2025:6612087. doi: 10.1155/prm/6612087 (PMC12453898; doi:10.1155/prm/6612087)
Supplement: Supporting Information — Additional supporting information can be found online in the Supporting Information section. [file 6612087.f1.docx]

**Supplementary Materials**

**Supplement 1. German version of the TEC scale.**

Skala der Behandlungserwartungen bei chronischen Schmerzen (TEC)

Die folgenden Fragen beziehen sich auf Ihre Erwartungen an die Behandlung Ihrer chronischen Schmerzen. Bitte kreuzen Sie für jede Frage das Feld an, das am ehesten dem entspricht,

(a) was Sie sich idealerweise von Ihrem Behandlungstermin erhoffen (was Sie sich in einer idealen Welt wünschen würden) und

(b) was Sie realistischerweise von Ihrem Behandlungstermin erwarten (was Ihrer Vermutung nach tatsächlich geschehen wird).

Bitte schätzen Sie auf einer 5 Punkte-Skala ein(1 = stimme überhaupt nicht zu, bis 5 = stimme vollkommen zu), inwieweit sie der Aussage in Bezug auf ihre idealen und vermuteten Erwartungen zustimmen.

|  | Was Sie sich **idealerweise** wünschen würden … | | | | | Was Ihrer Vermutung nach **realistischerweise** gesche-hen wird … | | | | |
| --- | --- | --- | --- | --- | --- | --- | --- | --- | --- | --- |
| 1. Meine Schmerzen werden deutlich zurückgehen. | 1 | 2 | 3 | 4 | 5 | 1 | 2 | 3 | 4 | 5 |
| 2. Ich werde Routinetätigkeiten (Kochen, Putzen, Körperpflege) besser ausführen können. | 1 | 2 | 3 | 4 | 5 | 1 | 2 | 3 | 4 | 5 |
| 3. Ich werde die Ursache für die Schmerzen erfahren. | 1 | 2 | 3 | 4 | 5 | 1 | 2 | 3 | 4 | 5 |
| 4. Ich werde einen klaren Schmerztherapieplan erhalten. | 1 | 2 | 3 | 4 | 5 | 1 | 2 | 3 | 4 | 5 |
| 5. Ich werde lernen, wie ich mit meinen Schmerzen umgehen kann. | 1 | 2 | 3 | 4 | 5 | 1 | 2 | 3 | 4 | 5 |
| 6. Ich werde mehr über meinen Schmerzzustand erfahren. | 1 | 2 | 3 | 4 | 5 | 1 | 2 | 3 | 4 | 5 |
| 7. Meine Stimmung wird sich deutlich verbes-sern. | 1 | 2 | 3 | 4 | 5 | 1 | 2 | 3 | 4 | 5 |
| 8. Der Schmerzspezialist wird meine Situation und alle damit einhergehenden Herausforderungen verstehen. | 1 | 2 | 3 | 4 | 5 | 1 | 2 | 3 | 4 | 5 |
| 9. Mein Schlaf wird sich deutlich verbessern. | 1 | 2 | 3 | 4 | 5 | 1 | 2 | 3 | 4 | 5 |

**Supplement 2. Diagnoses**

| ID | Diagnoses |
| --- | --- |
| 1 | R52.2,R51 |
| 2 | M25.56,M17.5,M54.5,M54.2,E66.07,E88.22,F32.0,M42.97 |
| 3 | F45.41, E80.2,G62.9,M54.16,M42.14,M15.0,M17.0 |
| 4 | NA |
| 5 | F45.41, R52.9, F33.0, R51 |
| 6 | G62.9,M79.64,E83.58,R20.8 |
| 7 | R52.2,G62.9,G62.88,M53.1 |
| 8 | R52.2,M48.02,M99.73 |
| 9 | G61.8,F45.41,G62.9,R07.2 |
| 10 | R52.2,U07.1,R53 |
| 11 | R52.2,G62.88,G62.9,M25.50,M54.2,M50.2 |
| 12 | G62.9,G44.2,M25.56,M25.55,R52.2 |
| 13 | G62.9,M79.65,M25.54,M54.86,M54.2,F45.41 |
| 14 | M25.56,F45.41,Z98.8,M79.65,M79.60 |
| 15 | NA |
| 16 | NA |
| 17 | NA |
| 18 | F54.41,M79.19,M54.89,Q65.8,Z96.6,F33.4 |
| 19 | F54.41,G44.2,M54.2,M54.10,M96.1,M17.0,M25.56,Z98.8,M25.51 |
| 20 | R52.2,G35.20,M25.86,M54.16 |
| 21 | F45.41,M54.4,M51.2,G55.1,M43.16 |
| 22 | M89.86,M76.8,R51 |
| 23 | G62.9, M48.02,M54.5,R52.2 |
| 24 | F54.41,M43.16,M54.86,M99.63,M48.06,M81,80,M42,16 |
| 25 | M54.86,M47.82,M48,06,G60.0,R52.2 |
| 26 | NA |
| 27 | R52.2,M54.3,M54.5,M47.2,G62.9,M79,67,M25.56 |
| 28 | G53.0,B02.9 |
| 29 | N80.3,R10.3,G62.9,Z98,8,F45.41 |
| 30 | R52.2,M76.67,M93.27,L90.5,G62.9,Z98.8 |
| 31 | M54.4,M54.12,F43.1,Z98.8,N80.8,R10.3,G44.2,G43.0 |
| 32 | F45.41,F43.2,M06.00,M15.8,G44.2,M25.51,M79.60,M54.5,I97.82 |
| 33 | F54.41,F40.01V,M54.4,S32.05 |
| 34 | R52.2,G35.20,G62.9,M67.48,M42.16,M46.1 |
| 35 | F45.51,M53.1,M54.1 |
| 36 | F45.51,F33.0,M79.64,G56.0;M25.56,M25.57,R51 |
| 37 | F45.51,M79.10,M51.2,Z96.65,Z96.66,M50.1,E66.86 |
| 38 | NA |
| 39 | F45.41,G58.0,R10.1,G62.9,M54.4,M06.99 |
| 40 | NA |
| 41 | F45.41,M53.1,M79.97,M54.5,G44.2,G43.0 |
| 42 | F45.41,F41.1,G62.88,M54.5,M79.19,M42.12,M48.02,M21.61,M25.56,M17.1,M19.01,M18.0 |
| 43 | NA |
| 44 | NA |
| 45 | F45.41,M79.10,M54.14,L90.5,Z98.8,N20.0,M79.63 |
| 46 | NA |
| 47 | F45.51,M79.10,M25.56,Z98.8 |
| 48 | NA |
| 49 | NA |
| 50 | R52.2,M17.1,M19.91,M79.61,S76.1,Z96.60,S46.0,R26.2,Z45.80 |
| 51 | G62.9,G56.2,G44.2,R10.4,M54.4,F45.41 |
| 52 | M16.9,M19.99,Z96.68;E80.1 |
| 53 | M79.65,Z98.8,R52.2,L90.5,R26.2 |
| 54 | R52.2,H93.1 |
| 55 | B02.2,G53.0 |
| 56 | F45.41,M96.1,M54.16,M79.27,M53.0,M53.1 |
| 57 | R10.3,N80.1,N80.3,N80.0,Z98.8,M54.80,M51.2,F45.41 |
| 58 | R52.2,G62.88 |
| 59 | R52.2,G62.9,M96.1,G43.1,Z89.1,G57.3,F45.41 |
| 60 | R52.2,M77.2,M18.9,G62.9 |
| 61 | G50.0,D32.0 |
| 62 | G62.9,R52.2 |
| 63 | B02.2,G53.0,M62.88,M54.2,R52.2 |
| 64 | M25.51,R52.2,M79.65,M25.56,M15.8,M15.8,G62.9,M54.2 |
| 65 | R52.2,G62.9,K10.8,R51 |
| 66 | F45.41,M15.9,M25.50,M17.9,M75.9,M54.5,M53.0,M79.19 |
| 67 | B02.2,G53.0,F45.41 |
| 68 | F45.41, S32.01,M80.88, Z96.64, M19.83 |
| 69 | R10.3, N80.8, F33.0, M54.86 |
| 70 | M79.19 |
| 71 | F45.41, M51.2, M25.56 |
| 72 | NA |
| 73 | F45.41, M54.4, M51.2, M47.86, M53.86 |
| 74 | F45.41, M79.97, M18.0, M25.56, M19.1 |
| 75 | NA |
| 76 | F45.41, G62.9, M54.5, M51.3, M16.0 |
| 77 | NA |
| 78 | F45.41, M54.5, M47.26, M25.25, G43.1, M53.0, G46.2 |
| 79 | F45.41, G44.3, M52.1, M53-0, M75.4, M54.4, G62.9 |
| 80 | NA |
| 81 | F45.41, M54.90, M42.04 |
| 82 | F45.41, M25.56, M54.5, M79.10, Z98.8, Z96.65, F33.0, G44.2 |
| 83 | F45.41, M54.4, M15.1 |
| 84 | F45.41, G82.18, G43.1, G44.2, M17.9, E66.0 |
| 85 | NA |
| 86 | NA |
| 87 | NA |
| 88 | M79.67, M19.07, M81.99, R52.2 |
| 89 | R52.2, G62.9, M79.65, G61.8 |
| 90 | F45.41, M25.51, M25.54, G57.1, M22.8, M47.82, M48.02, M51.2, R10.1 |
| 91 | F45.41, G62.9, M54.2, M48.02, G62.88, M17.0, M25.50, M79.10 |
| 92 | R52.2, R10.2, M79.65, M62.88, M54.2 |
| 93 | R39.8, R10.2, R52.2, M54.5 |
| 94 | F45.41, L40.5, M07.39, M07.30, L40.0, M25.50, R29.8, M54.2, G44.2 |
| 95 | M79.19, M53.1, M25.54, M25.50 |
| 96 | NA |
| 97 | R52.9, R52.2, R20.2, G62.88, M25.50, M79.70 |
| 98 | R52.2, G62.0 |
| 99 | R52.2, M79.65, M79.60, M80.00, M99.73, M43.16, R26.2, R29.6 |
| 100 | M54.4, G24.3, M25.51, M25.56, Z96.64, G56.0, R52.2 |
| 101 | T87.6, G62.9, G54.7, T84.8, M25.56 |
| 102 | F45.41, F32.8, F40.1, M54.80, M53.0, M16.1, M75.4, M25.56, M22.4, M22.8, M77.3 |
| 103 | G90.59, G62.9, G56.2, M79.62 |
| 104 | R52.9, M25.50, M54.80, M19.07, Z96.65, Z96.60, G62.9, G62.88, F45.41 |
| 105 | F45.41, M54.2, M25.50, L40.5, G44.2, M54.6, M54.5, R52.9 |
| 106 | G90.59 |
| 107 | R52.2, M79.60, U09.9, M79.19, M54.5, G44.2, G43.1 |
| 108 | M25.50, M54.82, M54.5, G44.2, M79.67, R52.2, U09.9, A69.2, H92.0 |
| 109 | R52.2, R07.2, N64.4, K10.8, M79.60, M54.86, G44.2 |
| 110 | R52.2, M54.2, M54.86, M25.51, M79.62, S46.0, S42.3, M17.0, M25.56, M25.57, S82.6 |
| 111 | F45.41, N94.8, R10.3, M79.60, M41.50, M48.02, M54.86, M41.0, R51, F41.0 |
| 112 | G57.1, F45.41 |
| 113 | R07.3, F45.41, M25.56 |
| 114 | F45.41, M48.06, M54.2, M25.56, M54.17, M16.0, M54.16, M42.16, I89.08, G60.3, R26.8, F41.2, F41.0 |
| 115 | M25.50, M79.19, F33.4 |
| 116 | G62.88, G62.9, F45.41, M48.06, M47.86, M51.2, M47.99, M79.10 |
| 117 | F45.41, G62.9, M79.66, M79.60, M84.16, R20.1 |
| 118 | F45.41, R52.1, G62.9, M54.2, M25.55, M25.51 |
| 119 | NA |
| 120 | F45.41, M96.1, M54.17, G57.6, Z96.65, M43.96, G43.9, G44.4, Z74.0 |
| 121 | F45.41, M13.80, M46.1, M13.5 |
| 122 | NA |
| 123 | NA |
| 124 | F45.41, G44.2, G43.1, M47.12, M50.2, M79.18, M62.98 |
| 125 | NA |
| 126 | F45.41, M79.19, M54.14, M25.54, M79.61, G44.2 |
| 127 | NA |
| 128 | F45.41, M53.0, M62.68, M25.51, M79.67 |
| 129 | NA |
| 130 | F45.41, R10.4, G62.9 |
| 131 | NA |
| 132 | NA |
| 133 | NA |
| 134 | F45.41, F45.40, M53.3, M54.5 |
| 135 | F45.41, G54.9, G43.9, M19.99, M54.17, M25.50 |
| 136 | F45.41, M79.81, M54.4, M25.50, F33.0, M47.10 |
| 137 | NA |
| 138 | F45.41, M79.19, M33.2 |
| 139 | NA |
| 140 | F45.41, M79.19, M62.98 |
| 141 | F45.41, M79.19, M54.2, M75.3, G44.2, M51.2 |
| 142 | F45.41, M54.2, M54.4, M43.16, G62.9, F32.1 |
| 143 | NA |
| 144 | F45.41, M54.4, M25.56, M25.50, G43.0, G44.2, F33.0 |
| 145 | NA |
| 146 | NA |
| 147 | NA |
| 148 | F45.41, M53.0, M54.4, M79.10, G43.1, G44.2, G44.4, F33.1 |
| 149 | NA |
| 150 | NA |
| 151 | F45.41, M25.56, G44.2, F33.0 |
| 152 | F45.41, M79.67, T93.2, G62.9, M54.4, M53.0 |
| 153 | M54.5, M43.17, M54.2 |
| 154 | F45.41, F45.40, F33.1, F44.1, M53.0, M54.4, G44.2, G44.1, M25.56 |
| 155 | F45.41, M50.2, M79.63, R20.2, M79.67, M76.6, M54.99, M46.1 |
| 156 | F45.41, M54.16, Z88.8, F19.1, F43.2 |
| 157 | F45.41, M54.4, M51.1, G58.1, M42.96, M96.1, Z96.67, M35.56, M23.51, G47.0 |
| 158 | F45.41, M13.80, M46.1, M13.5 |
| 159 | NA |
| 160 | NA |
| 161 | F45.41, G44.2, G43.1, M47.12, M50.2, M79.18, M62.98 |
| 162 | NA |
| 163 | F45.41, M79.19, M54.14, M25.54, M79.61, G44.2 |
| 164 | F45.41, M79.81, M54.4, M25.50, F33.0, M47.10 |
| 165 | NA |
| 166 | F45.41, M79.19, M33.2 |
| 167 | NA |
| 168 | F45.41, M79.19, M62.98 |
| 169 | F45.41, M79.19, M54.2, M75.3, G44.2, M51.2 |
| 170 | F45.41, M54.2, M54.4, M43.16, G62.9, F32.1 |
| 171 | NA |
| 172 | F45.41, M54.4, M25.56, M25.50, G43.0, G44.2, F33.0 |
| 173 | G90.59, G62.9, G56.2, M79.62 |
| 174 | R52.9, M25.50, M54.80, M19.07, Z96.65, Z96.60, G62.9, G62.88, F45.41 |
| 175 | F45.41, M54.2, M25.50, L40.5, G44.2, M54.6, M54.5, R52.9 |
| 176 | F45.41, M51.2, M25.56 |
| 177 | NA |
| 178 | F45.41, M54.4, M51.2, M47.86, M53.86 |
| 179 | F45.41, M79.97, M18.0, M25.56, M19.1 |
| 180 | NA |
| 181 | F45.41, G62.9, M54.5, M51.3, M16.0 |
| 182 | F45.51,M53.1,M54.1 |
| 183 | F45.51,F33.0,M79.64,G56.0;M25.56,M25.57,R51 |
| 184 | F45.51,M79.10,M51.2,Z96.65,Z96.66,M50.1,E66.86 |
| 185 | NA |
| 186 | F45.41,G58.0,R10.1,G62.9,M54.4,M06.99 |
| 187 | NA |
| 188 | F45.41, M54.5, M47.26, M25.25, G43.1, M53.0, G46.2 |
| 189 | F45.41, G44.3, M52.1, M53-0, M75.4, M54.4, G62.9 |
| 190 | F45.41, M54.4, M15.1 |
| 191 | F45.41, M54.90, M42.04 |
| 192 | F45.41, M25.56, M54.5, M79.10, Z98.8, Z96.65, F33.0, G44.2 |

**Supplement 3. Item response functions**

| **Predicted subscale** | **Ideal subscale** |
| --- | --- |
| 1. Meine Schmerzen werden deutlich zurückgehen. | |
| 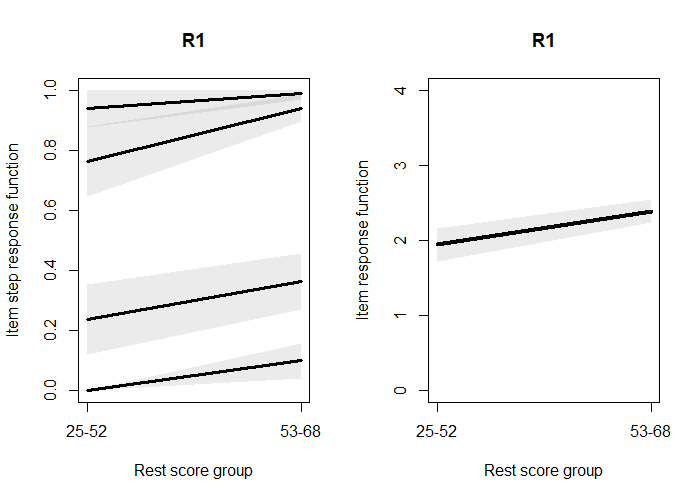 | 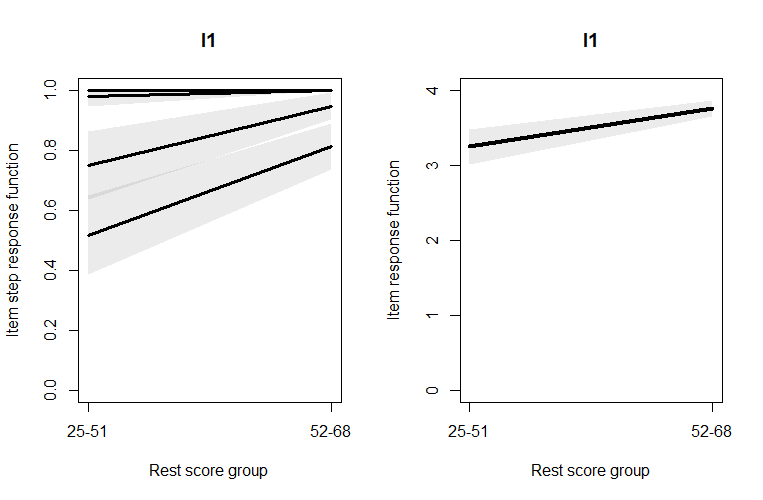 |
| 2. Ich werde Routinetätigkeiten (Kochen, Putzen, Körperpflege) besser ausführen können. | |
| 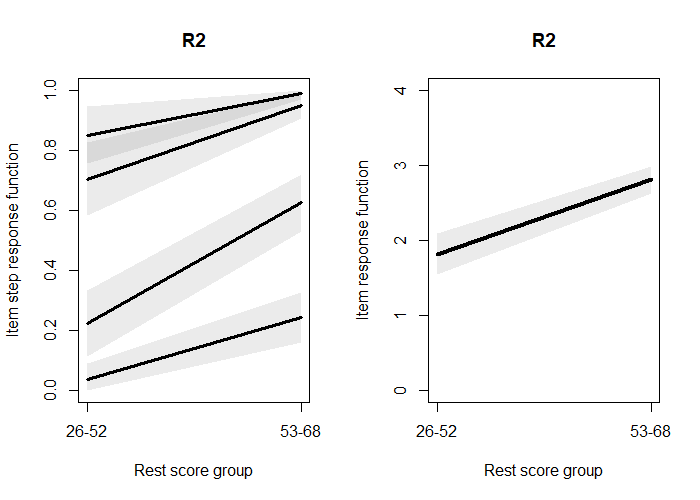 | 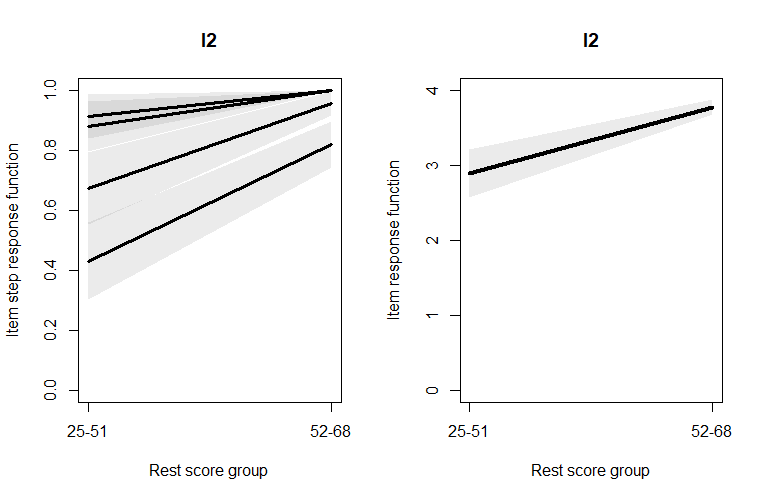 |
| 3. Ich werde die Ursache für die Schmerzen erfahren. | |
| 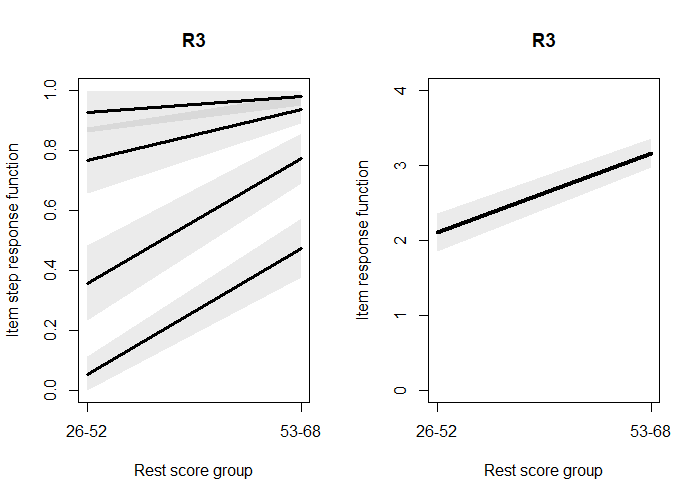 | 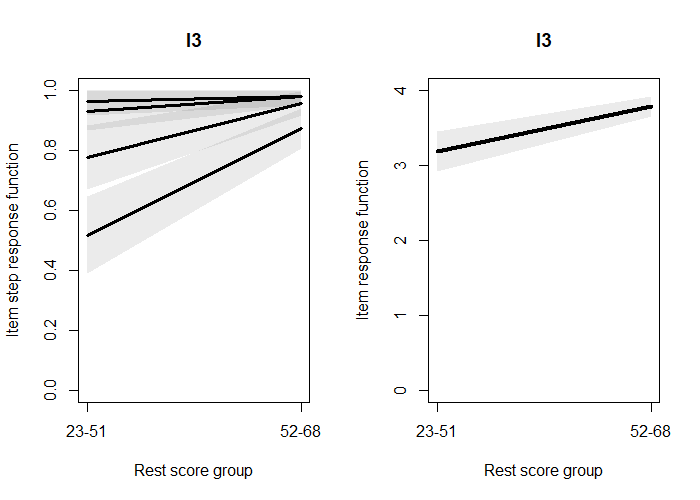 |
| 4. Ich werde einen klaren Schmerztherapieplan erhalten. | |
| 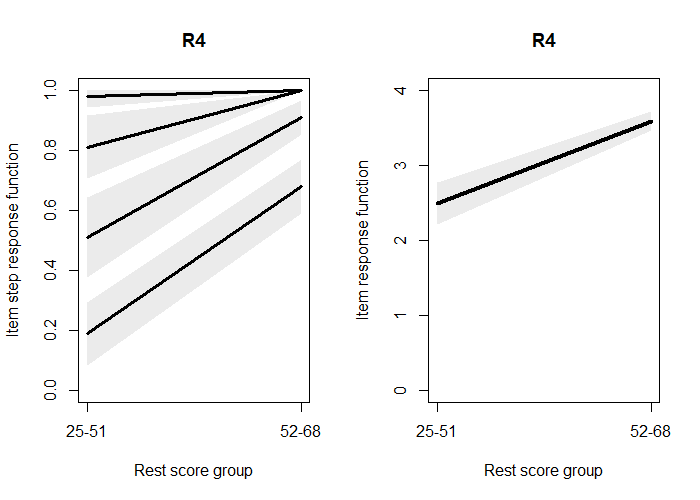 | 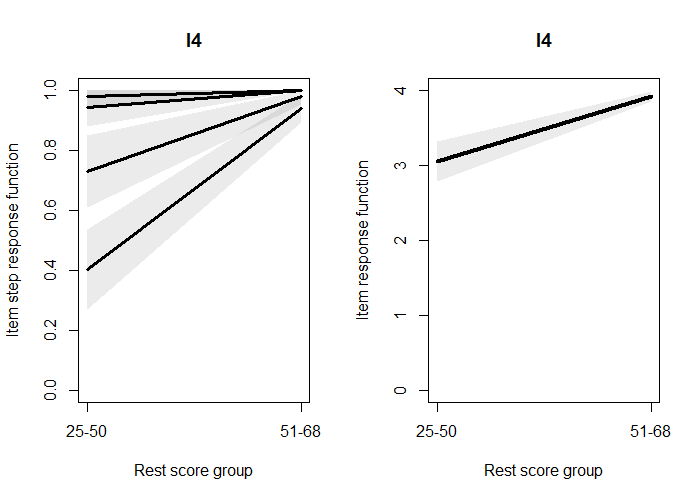 |
| 5. Ich werde lernen, wie ich mit meinen Schmerzen umgehen kann. | |
| 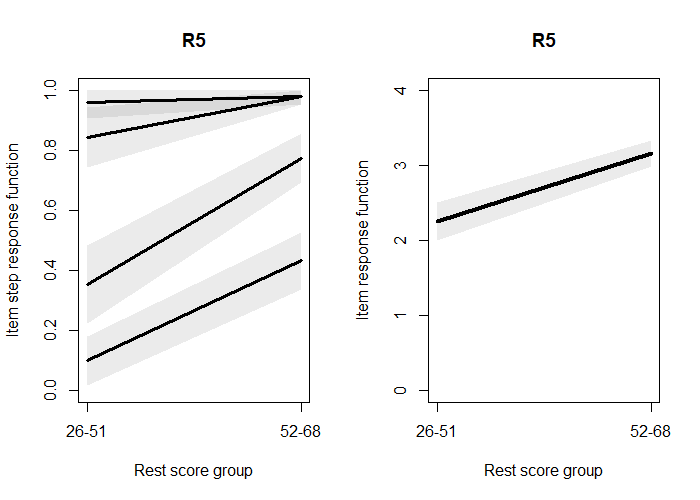 | 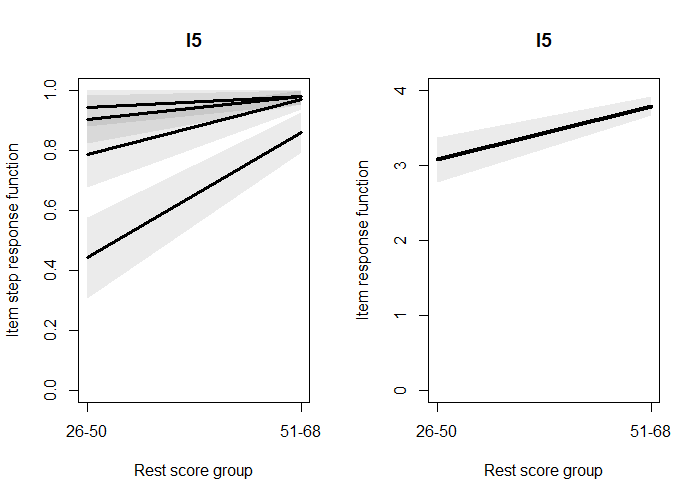 |
| 6. Ich werde mehr über meinen Schmerzzustand erfahren. | |
| 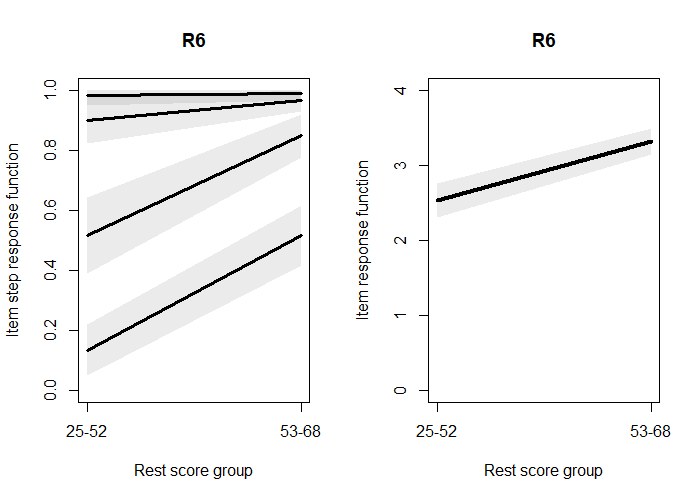 | 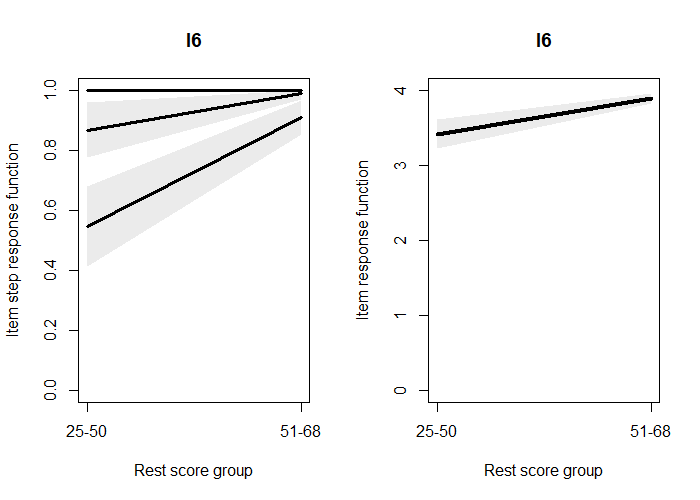 |
| 7. Meine Stimmung wird sich deutlich verbessern. | |
| 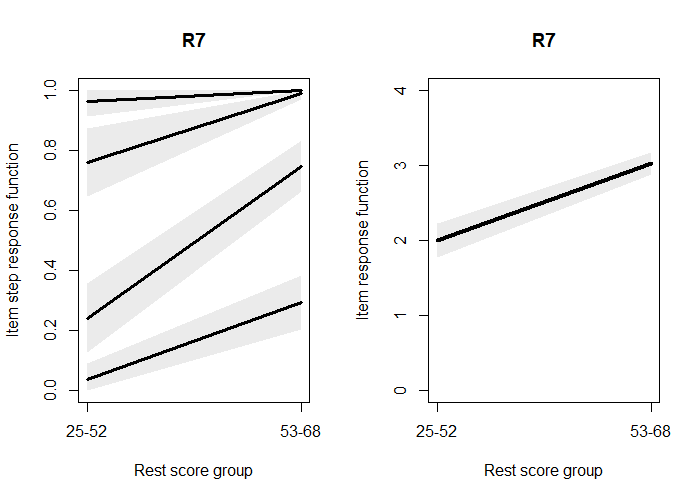 | 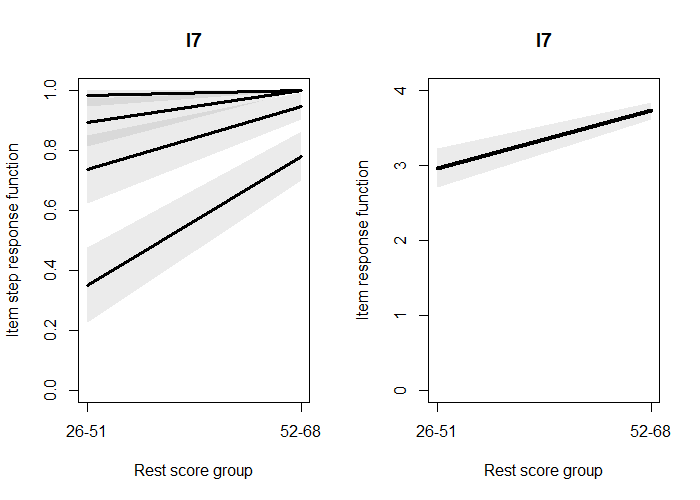 |
| 8. Der Schmerzspezialist wird meine Situation und alle damit einhergehenden Herausforderungen verstehen. | |
| 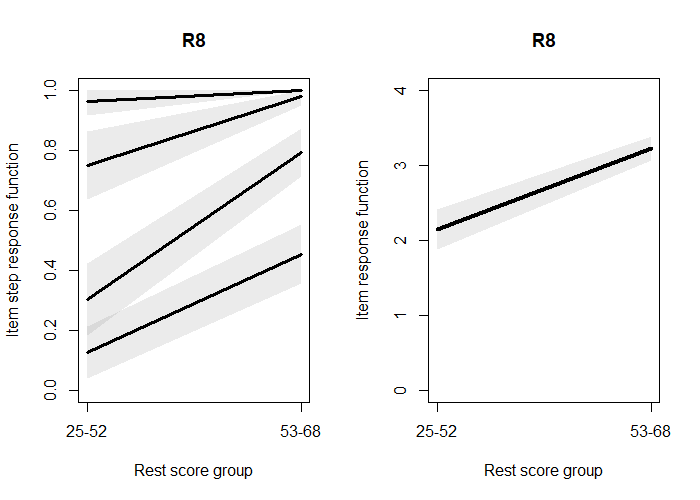 | 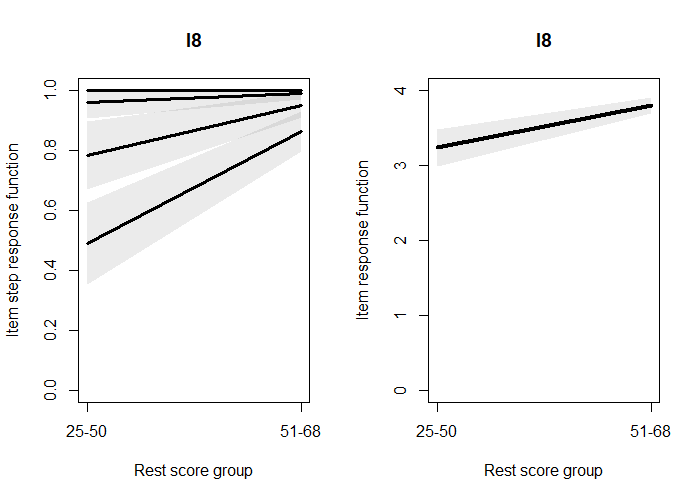 |
| 9. Mein Schlaf wird sich deutlich verbessern. | |
| 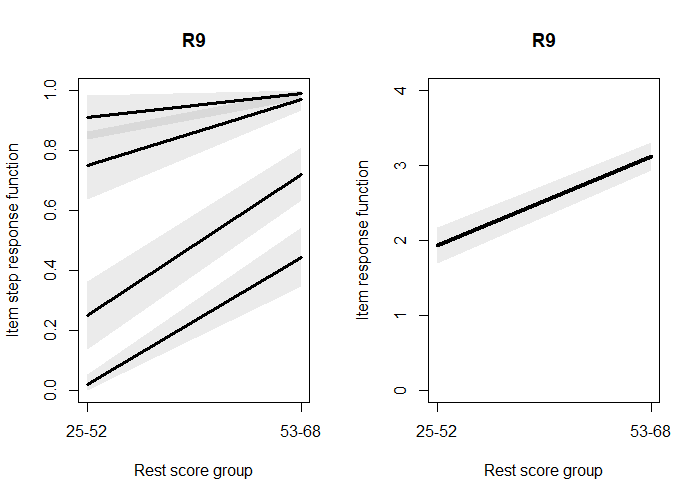 | 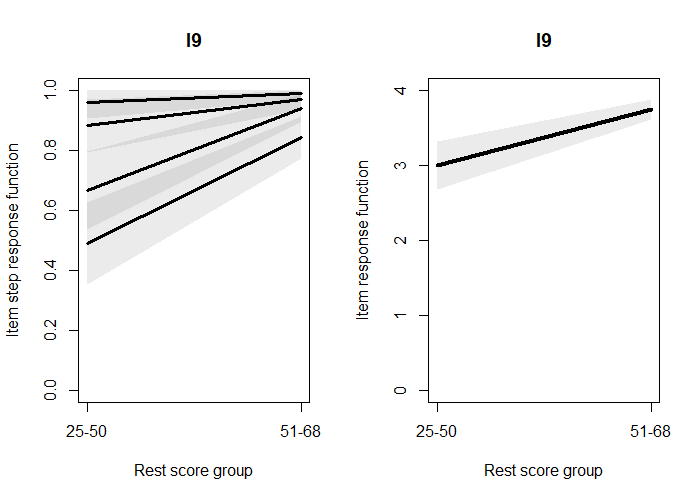 |

**Supplement 4. Scale properties for DASS and LOT-R**

Internal consistency for the external measures was satisfactory. In the present sample, the DASS-21 Depression sub-scale demonstrated excellent reliability (Cronbach’s α = .88; McDonald’s ω_h_ = .87, ω_t_ = .87), while the DASS-21 Anxiety sub-scale showed good reliability (α = .79; ω_h_ = .79, ω_t_ = .79). For the Life Orientation Test–Revised (LOT-R), internal consistency was acceptable for the Optimism sub-scale (α = .70; ω_h_ = .70, ω_t_ = .70) and slightly lower but still adequate for the Pessimism sub-scale (α = .67; ω_h_ = .66, ω_t_ = .66).
